# Classic Congenital Adrenal Hyperplasia Due To 21-hydroxylase Deficiency – the Next Disease Included in The Neonatal Screening Program in Poland

**DOI:** 10.34763/devperiodmed.20182202.197200

**Published:** 2018-06-30

**Authors:** Maria Ginalska-Malinowska

**Affiliations:** 1 Screening and Metabolic Diseases Department, Institute of Mother and Child, Warsaw, Poland

**Keywords:** congenital adrenal hyperplasia, CAH, 21-hydroxylase deficiency, neonatal screening, wrodzony przerost nadnerczy, WPN, niedobór 21-hydroksylazy, noworodkowe badania przesiewowe

## Abstract

Congenital adrenal hyperplasia (CAH) is a group of autosomal recessive disorders characterized by impaired cortisol synthesis. The most common form of CAH is caused by mutations in CYP21A, the gene encoding the adrenal steroid 21-hydroxylase enzyme. Deficiency of the enzyme leads to life-threatening adrenocortical insufficiency, which is not demonstrable during the first days of life. Additionally, some of the affected neonates have varying degrees of pathology of the external genitalia, classified as disorders of sex development (DSD). These make it difficult to recognize the sex of the affected neonate or lead to incorrect sex assignment.

CAH has been included in neonatal screening programs in many countries of the world since the late of 1970s. The main benefit of the screening is early diagnosis and prevention of neonatal mortality in children with salt-wasting CAH. Early recognition of the disease is also helpful in correct sex assignment of DSD neonates.

In 2016 Poland joined the group of countries which conduct neonatal screening for 21-hydroxylase deficient CAH and the condition is now included in the neonatal screening program. Therefore, it is possible to recognize the disease soon after birth in all Polish newborns and to start the prompt replacement steroid therapy. As the information on the suspicion or diagnosis of CAH in very young newborns has recently reached neonatologists, pediatricians, and general practitioners, and not only pediatric endocrinologists, the aim of this paper is to deliver the most necessary information on the disease to a wider group of doctors, not familiar with CAH.

## Streszczenie

Wrodzony przerost nadnerczy jest to dziedziczone autosomalnie recesywnie wrodzone zaburzenie wytwarzania kortyzolu w korze nadnerczy. Spowodowane jest najczęściej mutacją w genie CYP21A i wynikającym z tego niedoborem 21-hydroksylazy – jednego z enzymów niezbędnych do prawidłowej steroidogenezy. Brak lub niedobór 21-hydroksylazy prowadzi do zagrażającej życiu dziecka wrodzonej niewydolności nadnerczy, nie zawsze ujawnionej klinicznie w pierwszych kilku/kilkunastu dniach życia noworodka. Często również jest przyczyną wrodzonych zaburzeń rozwoju narządów płciowych, utrudniających określenie płci dziecka w momencie narodzin powodując, że jego płeć może być ustalona niewłaściwie.

Klasyczna forma wrodzonego przerostu nadnerczy spowodowanego niedoborem 21-hydroksylazy od początku lat 80-tych XX wieku jest stopniowo wprowadzana do programu noworodkowych badań przesiewowych w coraz większej liczbie krajów na świecie. Dzięki możliwości rozpoznania wrodzonego przerostu nadnerczy przed wystąpieniem objawów klinicznych zmniejsza się śmiertelność noworodków z najcięższą postacią choroby, przebiegającą z zespołem utraty soli. Wczesne rozpoznanie jest także istotne dla określenia płci dziecka w razie istniejących wątpliwości.

Od 2016 roku również Polska dołączyła do grona państw świata, w których wrodzony przerost nadnerczy należy do panelu noworodkowych badań przesiewowych. Dzięki temu już w pierwszych dniach po urodzeniu możliwe jest rozpoznanie choroby i wdrożenie odpowiedniego leczenia hormonalnego u wszystkich polskich noworodków. Z uwagi na fakt, że informacja o podejrzeniu / rozpoznaniu wrodzonego przerostu nadnerczy u kilkudniowych noworodków dociera teraz do szerszego grona lekarzy – pediatrów, neonatologów, lekarzy rodzinnych, a nie tylko endokrynologów dziecięcych, praca ta ma na celu przybliżenie zagadnienia choroby szerszemu gronu lekarzy pediatrów w Polsce.

## Introduction

Congenital adrenal hyperplasia (CAH) is a group of autosomal recessive disorders caused by the deficiency of the enzymes required for cortisol biosynthesis in the adrenal cortex [1, 2]. The most common defect (in more than 90% of CAH cases ) is 21-hydroxylase deficiency resulting from mutations or deletions in *CYP21A2*, the gene encoding steroid adrenal 21-hydroxylase. The frequency of this type of CAH is estimated at 1:10,000-1:20,000 live births [3]. The classic salt-wasting form of the disease is associated with life-threatening adrenal insufficiency in newborns and is reported in approximately 75-80% of the cases. In addition, CAH due to 21-hydroxylase deficiency is the most common cause of genital ambiguity in the newborn.

## Pathogenesis

**Fig. 1:**
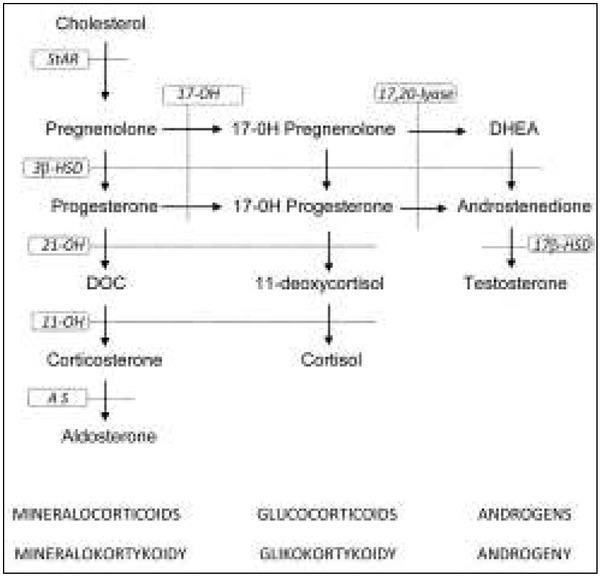
Schematic diagram of adrenal steroidogenesis Steroidogenic enzymes (in frames): StAR - steroidogenic acute regulatory protein 17-OH - 17α-hydroxylase/17,20-lyase 3β-HSD - 3β-hydroxysteroid dehydrogenase 21-OH - 21-hydroxylase 11-OH - 11β-hydroxylase AS - aldosterone synthase 17β-HSD - 17β-hydroxysteroid dehydrogenase Hormones: DOC - 11-deoxyorticosterone; DHEA – dehydroepiandrosterone; Ryc. 1. Schemat steroidogenezy nadnerczowej Enzymy steroidogenezy (w ramkach): StAR - steroidowe ostre białko regulatorowe 17-OH - 17α-hydroksylaza/17,20-liaza 3β-HSD - dehydrogenaza 3β-hydroksysteroidowa 21-OH - 21-hydroksylaza 11-OH - 11β-hydroksylaza AS - syntaza aldosteronowa 17β-HSD - dehydrogenaza 17β-hydroksysteroidowa Hormony: DOC - 11-deoksykortykosteron; DHEA – dehydroepiandrosteron;

**Fig. 2:**
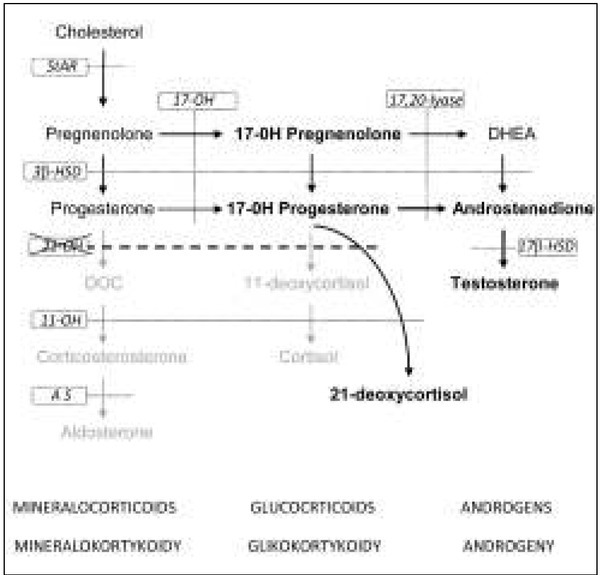
Schematic diagram of adrenal steroidogenesis in 21-hydroxylase deficiency Steroidogenic enzymes (in frames): StAR - steroidogenic acute regulatory protein 17-OH - 17α-hydroxylase/17,20-lyase 3β-HSD - 3β-hydroxysteroid dehydrogenase 21-OH - 21-hydroxylase 11-OH - 11β-hydroxylase AS - aldosterone synthase 17β-HSD - 17β-hydroxysteroid dehydrogenase Hormones: DOC - 11-deoxyorticosterone; DHEA – dehydroepiandrosterone Ryc. 2. Schemat steroidogenezy nadnerczowej przy niedoborze 21-hydroksylazy Enzymy steroidogenezy (w ramkach): StAR - steroidowe ostre białko regulatorowe 17-OH - 17α-hydroksylaza/17,20-liaza 3β-HSD - dehydrogenaza 3β-hydroksysteroidowa 21-OH - 21-hydroksylaza 11-OH - 11β-hydroksylaza AS - syntaza aldosteronowa 17β-HSD - dehydrogenaza 17β-hydroksysteroidowa Hormony: DOC - 11-deoksykortykosteron; DHEA – dehydroepiandrosteron

Steroid hormone synthesis begins with cholesterol – both in the adrenal cortex and in the gonads. Three classes of adrenal hormones are produced from cholesterol: mineralocorticoids, glucocorticoids and androgens (sex hormones). The main mineralocorticoid of the adrenal zona glomerulosa is aldosterone, and cortisol is the main glucocorticoid formed in the adrenal zona fasciculata in humans (Figure 1). Some androgens (testosterone, androstenedione, dehydroepiandrosterone – DHEA) are produced in the adrenal zona reticularis in both sexes.In 21-hydroxylase deficiency cortisol production is decreased, therefore the corticotrophin-releasing hormone (CRH) from the hypothalamus and adrenocorticotropic hormone (ACTH) from the pituitary are increased via a negative feedback loop. Consequently, the adrenal glands become hyperplastic and they produce an excess of adrenal androgens that do not require 21 -hydroxylase for their synthesis. In the zona fasciculata there can be both an overproduction of 17-hydroxyprogesterone (17-OHP), the hormone prior to the enzymatic block, and a deficiency of the hormones distal to the disordered enzymatic step (11-deoxycortisol and cortisol) (Figure 2). Some highly increased 17-OHP via 11β-hydroxylase is metabolized to 21-deoxycortisol, which is used as a marker of 21-hydroxylase deficiency. Most cases of the classic form of CAH due to 21-hydroxylase deficiency also have a significantly decreased synthesis of aldosterone. These patients cannot maintain sodium balance, presenting with a potentially lethal salt-wasting crisis soon after birth. Adrenal steroidogenesis disorders of classic CAH forms start in the early prenatal period. Excessive androgens lead to the virilization of affected females *in utero* and genital ambiguity is presented at birth [1, 3, 4, 5]. In contrast, male newborns with CAH do not show ambiguous genitalia and therefore have a higher risk of adrenal crisis if not promptly diagnosed and treated.

## Clinical signs and symptoms

Clinical presentation of the disease depends on the patient’s genetic sex and on the severity of 21-hydroxylase deficiency. Clinically, early-onset classical CAH consists of two variants: the salt wasting (SW) form and the non-salt wasting/simple virilizing (SV) form. The majority of patients (75-80%) demonstrate the SW form with both cortisol and aldosterone production decreased. Soon after birth, clinical signs and symptoms may be nonspecific: poor appetite, lethargy, failure to thrive, vomiting. After a few days, more characteristic features are shown: dehydration, hyponatremia, hyperkalemia and hypovolemic shock. A less severe SV form with no severe electrolyte abnormalities is found in the remaining 20-25% of the patients with classic CAH.

Adrenal androgen production is excessive in both classic CAH forms in girls and boys but only affected females are virilized at birth, ranging from slight clitoromegaly to complete masculinization of the external genitalia. In most severely virilized females usually male sex is mistakenly assigned. Most newborn boys with 21-hydroxylase deficiency show normal appearance of the external genitalia at birth, and they have no clinical signs which could alert physicians to make a prompt diagnosis of CAH. Continued excessive adrenal androgen production in untreated SV patients (both boys and girls) causes rapid somatic growth, marked advancement of bone age, early development of pubic hair, as well as penis size increase in boys, or progressive clitoromegaly in girls.

## Diagnosis

On the clinical basis alone, an early diagnosis of the disease is almost impossible in a male affected infant and in a completely virilized CAH female newborn (assigned as male). Morbidity and mortality are high in this groups of patients [1, 3]. Only in newborns with ambiguous genitalia is the diagnosis of classic CAH strongly suspected.

The most characteristic biochemical abnormality in classic 21 -hydroxylase deficiency is elevation of 17-OHP, the main substrate for the enzyme. In CAH-affected patients basal serum 17-OHP values usually exceed 100 ng/ ml [1, 3, 4]. It is important to remember that 17-OHP levels are normally higher at the time of birth and decrease over the course of several days, whereas in affected infants, 17-OHP levels rise over time. Premature, low-birth-weight or stressed and sick newborns often have higher 17-OHP levels with no inborn errors in steroidogenesis [2, 6]. Serum concentrations of testosterone in girls and androstenedione in boys and girls are also elevated in affected infants.

What is typical of salt-wasting adrenal cortical failure is hyponatremia, hyperkalemia, hypovolemia and elevated levels of serum urea nitrogen. However, these symptoms are usually not present before 6-7 days of life.

The diagnosis of classic CAH is essential as soon as possible, before the onset of adrenal crisis in newborns. Whereas affected female infants usually have ambiguous genitalia, male infants appear normal and are thus more difficult to diagnose. Therefore, newborn CAH screening has been proposed, first in the US in 1977 and then it was initiated in many countries worldwide [7, 8]. CAH due to 21-hydroxylase deficiency can be detected in newborn screening programs by measuring the amount of 17-OHP in dried blood spots collected on filter paper cards from infants soon after birth.

After identifying infants with 21-hydroxylase deficiency, presymptomatic treatment can be started to prevent salt-wasting crises. Screening significantly reduces morbidity and mortality, particularly among male infants, and prevents male sex assignment in affected females. Since 2016 Poland has joined the countries performing neonatal screening for 21 -hydroxylase deficient CAH infants. Research and observation results will be the topic of a subsequent paper.

## Treatment

The goal of CAH glucocorticoid therapy is to reduce excessive adrenal androgens and to replace those hormones that are deficient. The administration of glucocorticoids reduces ACTH production and reverses adrenal hyperplasia with coexisting androgen excess. Hydrocortisone remains the therapy of choice in growing patients with classic CAH. Most often, the standard dose in maintenance therapy is in the range of 10-15 mg/ m^2^/day administered orally in 3 divided doses (infants usually require 2.5-5 mg 3 times/ day, and children 5-10 mg 3 times/ day). Hydrocortisone doses must be individualized by monitoring growth, bone age, and hormonal levels.

All classic CAH patients should be treated with mineralocorticoids (fludrocortisone) in the newborn period [3, 4]. Dosage requirements in early infancy range from 0.05–0.30 mg/day, whereas typical maintenance doses are 0.05-0.2 mg/day, depending on the sodium intake. It is extremely important to monitor blood pressure in all the patients who are receiving mineralocorticoid supplementation.

In infants with severe salt-wasting symptoms, treatment should include immediate i.v. administration of isotonic fluids (at least 20 ml/kg) and hydrocortisone i.v. bolus in much higher doses (100 mg/m^2^, in infants usually 25 mg). Hydrocortisone should then be given in divided doses every 4 to 6 hours until the child is stable. The dose of hydrocortisone can be tapered over a few days until the maintenance dose is attained [3, 4].

Additionally, the surgical treatment of virilized external genitalia is required in all affected females with classic CAH. Clitoroplasty with vaginal reconstruction is performed as a single-stage procedure usually in the first year of life.
